# Chitosan regulates metabolic balance, polyamine accumulation, and Na^+^ transport contributing to salt tolerance in creeping bentgrass

**DOI:** 10.1186/s12870-020-02720-w

**Published:** 2020-11-04

**Authors:** Wan Geng, Zhou Li, Muhammad Jawad Hassan, Yan Peng

**Affiliations:** Department of Grassland Science, College of Animal Science and Technology, Sichuan Agricultural University, Chengdu, 611130 China

**Keywords:** Ion transportation, Sugar metabolism, SOS pathway, Amino acids, Osmotic adjustment, Compartmentalization, Gene expression

## Abstract

**Background:**

Chitosan (CTS), a natural polysaccharide, exhibits multiple functions of stress adaptation regulation in plants. However, effects and mechanism of CTS on alleviating salt stress damage are still not fully understood. Objectives of this study were to investigate the function of CTS on improving salt tolerance associated with metabolic balance, polyamine (PAs) accumulation, and Na^+^ transport in creeping bentgrass (*Agrostis stolonifera*).

**Results:**

CTS pretreatment significantly alleviated declines in relative water content, photosynthesis, photochemical efficiency, and water use efficiency in leaves under salt stress. Exogenous CTS increased endogenous PAs accumulation, antioxidant enzyme (SOD, POD, and CAT) activities, and sucrose accumulation and metabolism through the activation of sucrose synthase and pyruvate kinase activities, and inhibition of invertase activity. The CTS also improved total amino acids, glutamic acid, and γ-aminobutyric acid (GABA) accumulation. In addition, CTS-pretreated plants exhibited significantly higher Na^+^ content in roots and lower Na^+^ accumulation in leaves then untreated plants in response to salt stress. However, CTS had no significant effects on K^+^/Na^+^ ratio. Importantly, CTS enhanced salt overly sensitive (SOS) pathways and also up-regulated the expression of *AsHKT1* and genes (*AsNHX4, AsNHX5*, and *AsNHX6*) encoding Na^+^/H^+^ exchangers under salt stress.

**Conclusions:**

The application of CTS increased antioxidant enzyme activities, thereby reducing oxidative damage to roots and leaves. CTS-induced increases in sucrose and GABA accumulation and metabolism played important roles in osmotic adjustment and energy metabolism during salt stress. The CTS also enhanced SOS pathway associated with Na^+^ excretion from cytosol into rhizosphere, increased *AsHKT1* expression inhibiting Na^+^ transport to the photosynthetic tissues, and also up-regulated the expression of *AsNHX4*, *AsNHX5*, and *AsNHX6* promoting the capacity of Na^+^ compartmentalization in roots and leaves under salt stress. In addition, CTS-induced PAs accumulation could be an important regulatory mechanism contributing to enhanced salt tolerance. These findings reveal new functions of CTS on regulating Na^+^ transport, enhancing sugars and amino acids metabolism for osmotic adjustment and energy supply, and increasing PAs accumulation when creeping bentgrass responds to salt stress.

**Supplementary Information:**

The online version contains supplementary material available at 10.1186/s12870-020-02720-w.

## Background

Crop growth and productivity is severely affected by salt stress all over the world. Plants uptake large amount of sodium ions (Na^+^) in cells resulting in the ionic imbalance under salt stress [1]. Excessive accumulation of Na^+^ leads to tissue necrosis and early aging of blades due to inhibition in protein synthesis, enzyme reactions, and photosynthesis [2–4]. Plants mediate Na^+^ concentration depending on the salt overly sensitive (SOS) pathways, Na^+^/H^+^ antiporters (NHX), and high-affinity Na^+^/K^+^-permeable transporter (HKT) in vacuoles and plasma membranes [5–7]. *SOS1* encodes a plasma membrane Na^+^/H^+^ antiporter which extrudes Na^+^ from the cytosol into the rhizosphere and is also involved in the long-distance transport of Na^+^ [8, 9]. *SOS3* and *SOS2* are necessary for the synthesis of *SOS1* mRNA and protein under salt stress and jointly participate in the regulation of *SOS1* phosphorylation [10]. In addition, the *SOS2* and *SOS3* may also regulate activities of H^+^-ATPase, H^+^-PPase, and NHX in the vacuolar membrane [11]. NHX mediates the exchange of Na^+^/H^+^ and K^+^/H^+^, thus affecting salt tolerance and potassium ions (K^+^) nutrition [12, 13]. It has been reported that *NHXs* overexpression can promote the efflux of excessive Na^+^ from cytoplasm and separate excess Na^+^ into vacuoles, resulting in increased salt tolerance in different plant species [14–16]. HKT1 plays an important role in retrieving Na^+^ from the xylem sap, thereby inhibiting Na^+^ transport to the photosynthetic tissues [7]. H^+^-ATPase or H^+^-PPase hydrolyzes ATP or PPi in the cytoplasm to pump H^+^ into the vacuole, which generates the electrochemical gradient and a proton motive force for the expulsion and separation of Na^+^ in cells [16–18]. Improved gene expression encoding H^+^-PPase could be associated with enhanced salt tolerance in transgenic *Lotus corniculatus* and alfalfa (*Medicago sativa*) [19, 20].

Sugars metabolism is regarded as a common response under different abiotic stresses [21]. Previous studies have shown that sucrose metabolism is one of key regulatory systems that confer tolerance to abiotic stresses, such as drought, high temperature and salt stress [22–24]. Chitosan (CTS) is a polycationic polysaccharide obtained by de-acetylation of chitin and also widely exists in plant species. In recent years, the CTS has been widely used in agricultural production as an exogenous additive substance being safe and cheap. The CTS exhibits positive effects on plant growth and tolerance to abiotic and biotic stresses [25, 26]. The study of Zhang et al. [27] found that CTS regulated a range of metabolic pathways including carbon and nitrogen metabolism in wheat (*Triticum aestivum*) leaves, thereby promoting plant growth. Exogenous CTS application effectively alleviated drought damage in white clover (*Trifolium repens*) through enhancing sucrose, mannose, and fructose accumulation in leaves [28]. The CTS also plays positive role in regulating salt tolerance in plants. For example, the CTS improved salt tolerance of maize (*Zea mays*) seedlings associated with enhancement in photosynthesis, glycolysis, and nitrogen assimilation [29]. With increasing salt stress, safflower (*Carthamus tinctorius*) and sunflower (*Helianthus annuus*) seed priming with CTS significantly improved seeds germination and antioxidant enzymes catalase (CAT) and peroxidase (POD) activities to mitigate oxidative damage in seedings [30]. Although these previous studies demonstrated that the CTS alleviated salt stress associated with some physiological effects such as antioxidant capacity, photosynthesis, and nitrogen assimilation, CTS-induced salt tolerance in relation to sugar and amino acid metabolism, polyamines (PAs) accumulation, and Na^+^ transport is still not completely elucidated in plants.

The turfgrass used in the sports industry needs higher frequency of maintenance and management than it is used in other area. A long-term use of chemicals, fertilizers, pesticides, and reclaimed water accelerates the soil salinization leading to the decline in turf quality and the increase in management difficulty and maintenance cost [31–33]. Creeping bentgrass (*Agrostis stolonifera*) is one of the most important turfgrasses and used extensively in the sports industry including golf courses and tennis lawns because of its high turf quality and creeping growth characteristics, however, salt tolerance of creeping bentgrass has been ranked as moderately sensitive [34]. Objectives of this study were to examine physiological effects of CTS on regulating water balance, antioxidant capacity, and photosynthesis salt stress. This study further hypothesizes that CTS-induced salt tolerance could be related to metabolite accumulation, changes in endogenous PAs, and Na^+^ transport in leaf and root. Current study provides a new and comprehensive insight into complex mechanism of CTS-regulated salt tolerance in plants.

## Results

### Effects of CTS on water status and photosynthesis in leaf

**Fig. 1:**
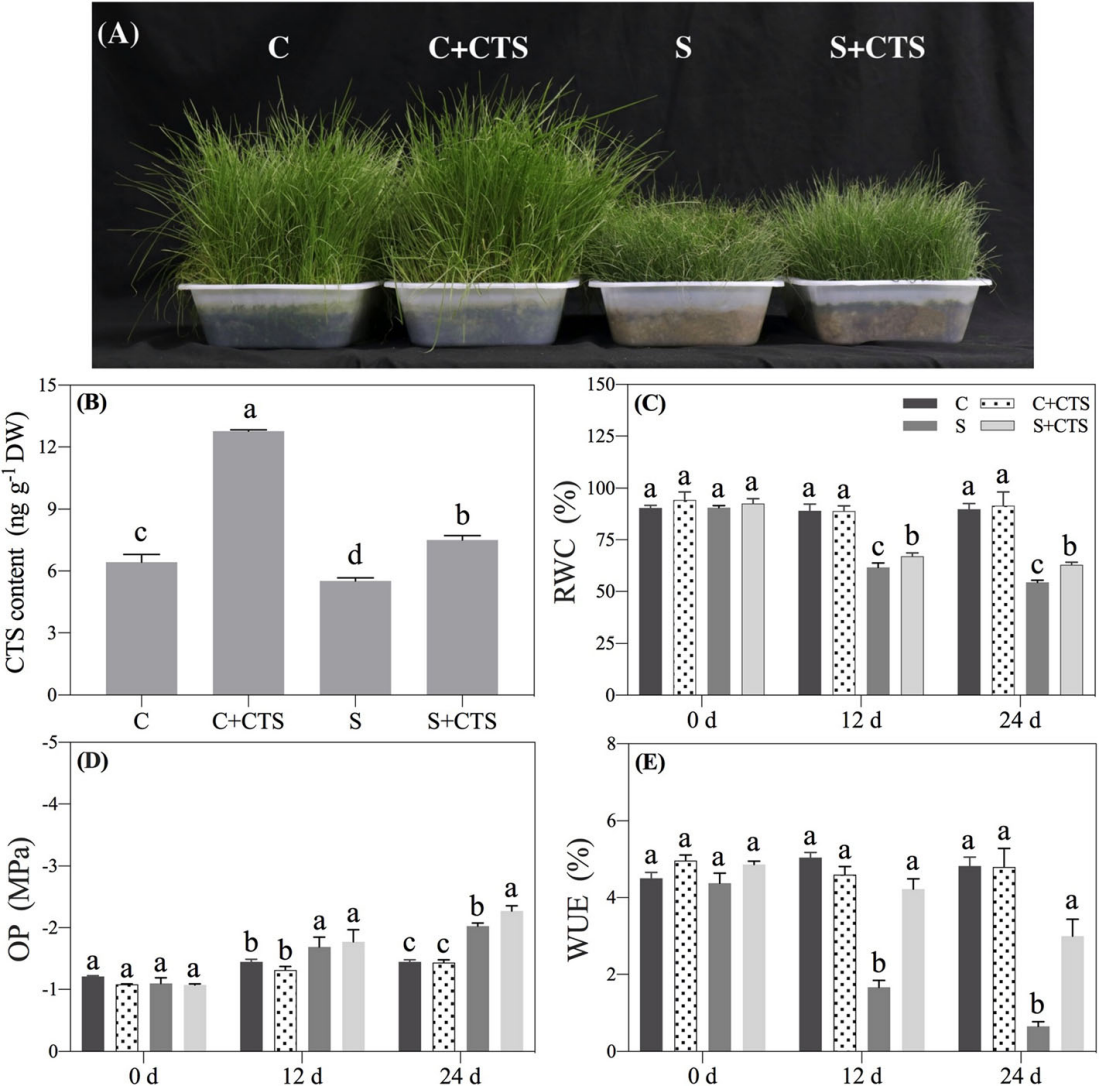
Changes in (**a**) phenotypes at 24 d, (**b**) CTS content at 24 d, (**c**) relative water content (RWC), (**d**) osmotic potential (OP), and (**e**) water use efficiency (WUE) in leaves of creeping bentgrass affected by the application of chitosan (CTS) under normal and salt stress conditions. Bars represent standard errors. Same letters above columns indicate no significant difference at a given day of treatment (*n* = 4, and *p* ≤ 0.05). C, control; C + CTS, control pretreated with CTS; S, salt stress; S + CTS, salt-stressed plants pretreated with CTS

**Fig. 2:**
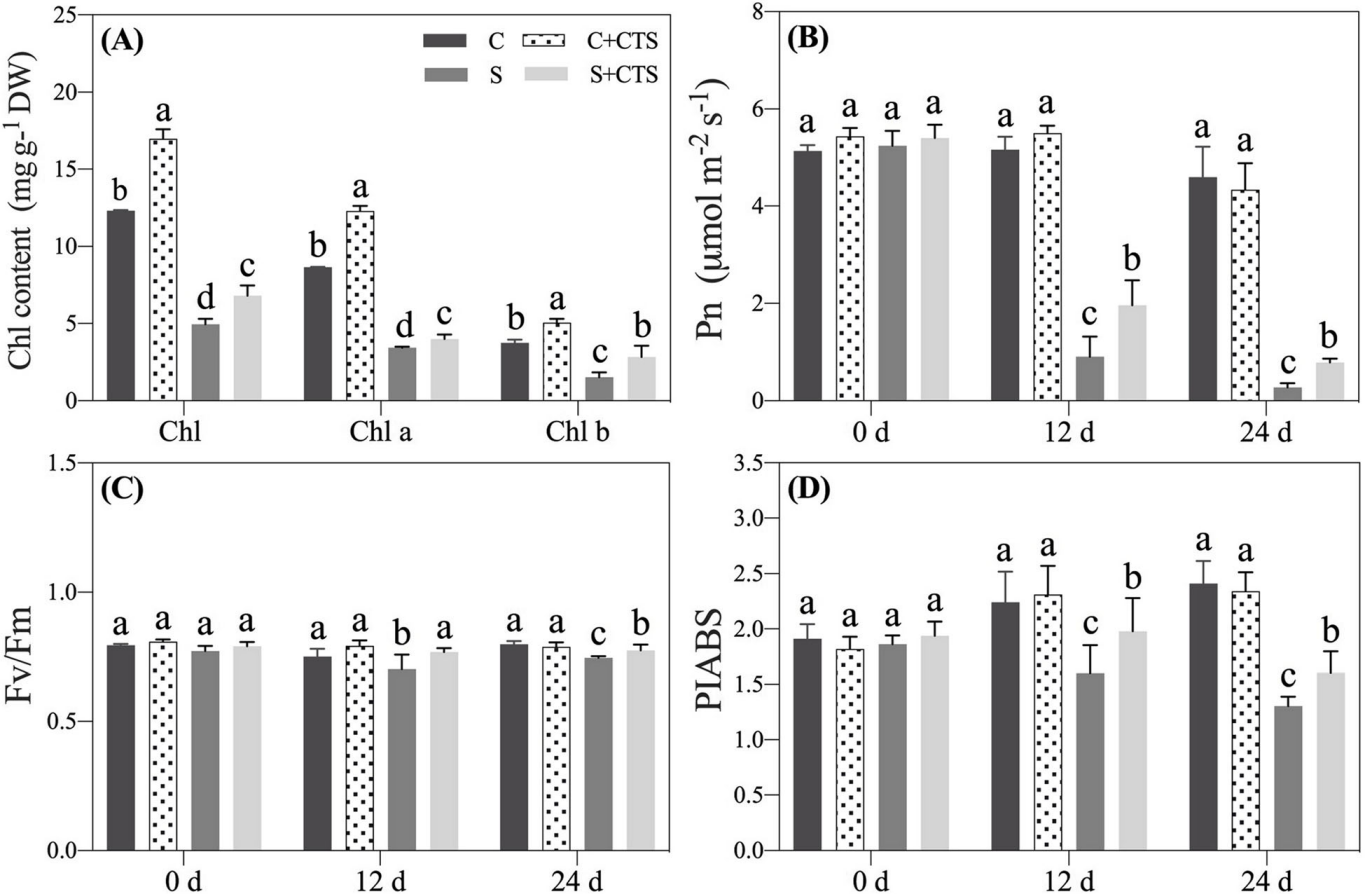
Changes in (**a**) chlorophyll (Chl) content at 24 d, (**b**) net photosynthesis rate (Pn), (**c**) photochemical efficiency (Fv/Fm), and (**d**) performance index on absorption basis (PIABS) in leaves of creeping bentgrass affected by the application of chitosan (CTS) under normal and salt stress conditions. Bars represent standard errors. Same letters above columns indicate no significant difference at a given day of treatment (*n* = 4, and *p* ≤ 0.05). C, control; C + CTS, control pretreated with CTS; S, salt stress; S + CTS, salt-stressed plants pretreated with CTS

To avoid salt shock and acclimate to salt stress, a successive increasing salt concentration (100 mmol/L NaCl solution for 4 days, 150 mmol/L NaCl solution for another 4 days, and 200 mmol/L NaCl for 16 days.) was applied in this study. Plant growth was inhibited under salt stress, but CTS-treated plants maintained better growth than untreated plants under salt stress (Fig. 1a). Salt stress significantly decreased CTS content, whereas exogenous CTS application improved CTS accumulation in leaves under normal condition and salt stress (Fig. 1b). Salt stress led to 30.70%, 16.81%, or 67.06% decrease in relative water content (RWC), osmotic potential (OP), or water use efficiency (WUE) in leaves at 12 d, respectively (Fig. 1c-e). CTS-treated plants had 15.65% increase of RWC and four times higher WUE than untreated plants at 24 d of salt stress (Fig. 1c and e). The OP indicates the potential of water movement between two regions associated with the water potential in cells. There was a significant difference in OP between CTS-treated and untreated plants at 24 d of salt stress (Fig. 1d). The application of CTS effectively alleviated salt-induced decline in chlorophyll (Chl), Chl a, and Chl b content in leaves (Fig. 2a). CTS-treated plants showed significantly higher net photosynthesis rate (Pn) than untreated plants at 12 or 24 d of salt stress (Fig. 2b). The performance index on absorption basis (PIABS) is a comprehensive photosynthetic index indicating the health status of leaves. For changes in photochemical efficiency (Fv/Fm) and PIABS, exogenous CTS did not affect these two parameters at 0, 12, and 24 d of normal condition, but significantly improved Fv/Fm and PIABS in leaves at 12 and 24 d of salt stress (Fig. 2c and d).

### Effects of CTS on antioxidant capacity in leaf

**Fig. 3:**
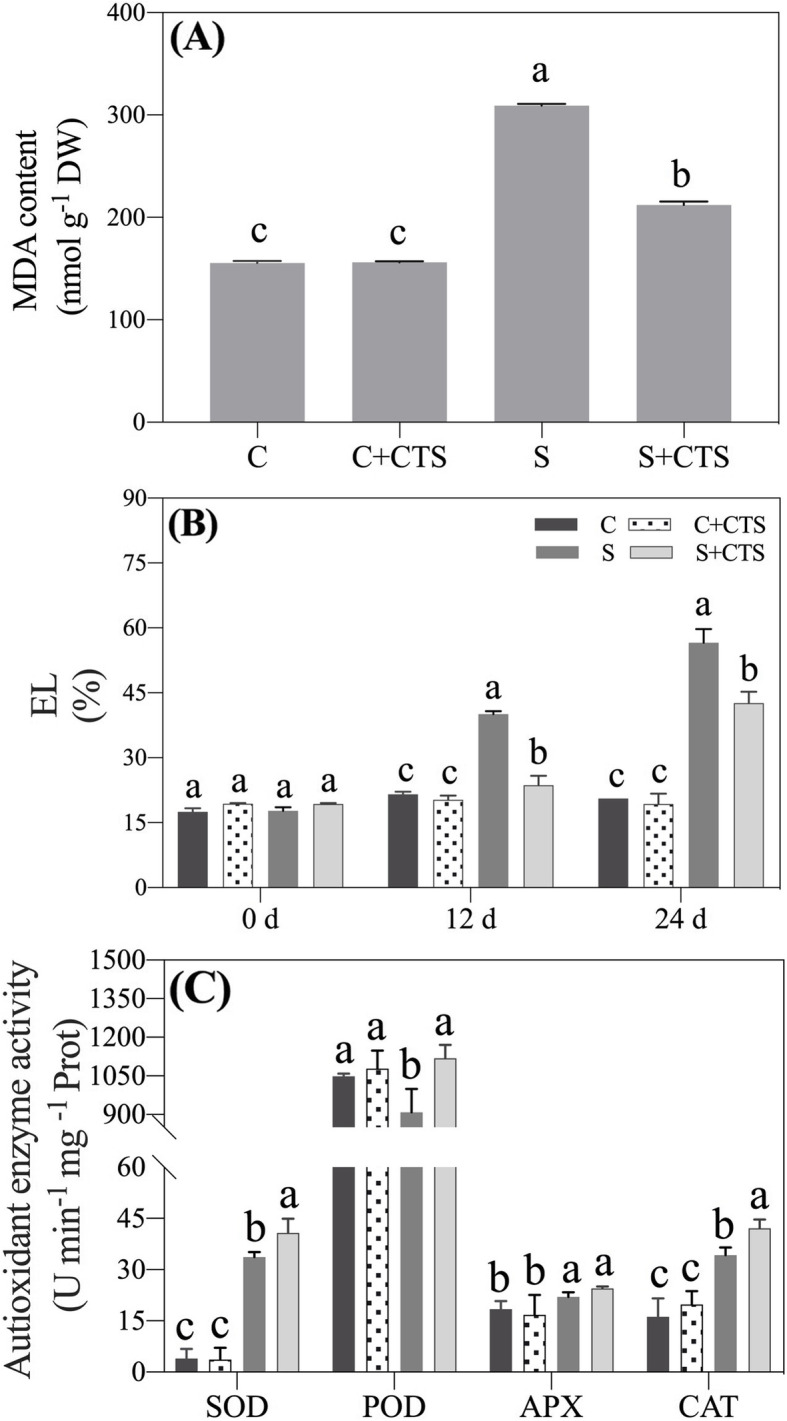
Changes in (**a**) malondialdehyde (MDA) content at 24 d, (**b**) electrolyte leakage (EL), and (**c**) antioxidant enzyme activities (SOD, superoxide dismutase; POD, peroxidase; APX, ascorbate peroxidase; CAT, catalase) in leaves of creeping bentgrass at 24 d affected by the application of chitosan (CTS) under normal and salt stress conditions. Bars represent standard errors. Same letters above columns indicate no significant difference (*n* = 4, and *p* ≤ 0.05). C, control; C + CTS, control pretreated with CTS; S, salt stress; S + CTS, salt-stressed plants pretreated with CTS

Malondialdehyde (MDA) is a key parameter related to lipid peroxidation level and electrolyte leakage (EL) reflects the degree of cell membrane stability. The MDA content increased significantly in leaves under salt stress. CTS pretreatment did not cause significant changes in MDA content under normal condition, but CTS-pretreated plants exhibited 31.37% decrease in MDA content than untreated plants at 24 d of salt stress (Fig. 3a). The EL gradually increased during salt stress. EL level was significantly lower in CTS-treated plants than that in untreated plants at 12 and 24 d of salt stress (Fig. 3b). For antioxidant enzyme activities, salt stress significantly increased superoxide dismutase (SOD), ascorbate peroxidase (APX), and CAT activities, but decreased POD activity in leaves of creeping bentgrass without CTS application (Fig. 3c). CTS-pretreated plants exhibited 17.24%, 18.80%, or 18.62% increase in SOD, POD, or CAT activity than untreated plants under salt stress, respectively. Exogenous CTS had no significant effects on POD activity under normal and stress conditions (Fig. 3c).

### Effect of CTS on sugars, amino acids, and energy metabolism in leaf

Salt stress significantly enhanced sucrose, glucose, and fructose accumulation in plants (Table 1). The ‘S + CTS’ treatment had 75.98% increase in sucrose content than the ‘S’ treatment (Table 1). CTS-pretreated plants exhibited significantly lower glucose and fructose content than untreated plants under salt stress (Table 1). Salt stress activated sucrose synthase (SS) and sucrose phosphate synthase (SPS) activities, but significantly inhibited invertase activity as showed in Table 1. CTS-pretreated plants demonstrated 33.04% increase in SS activity and also had 16.79% and 47.04% decreases in invertase and SPS activities than untreated plants under salt stress, respectively (Table 1). Total amino acids content (TAA), free proline, and glutamic acid (Glu) content increased significantly in leaves under salt stress, however, a significant decrease in γ-aminobutyric acid (GABA) content was observed under salt stress (Table 1). CTS-treated plants exhibited 19.84%, 6.79%, and 29.48% increases in TAA, glutamic acid, and GABA content compared with untreated plants under salt stress (Table 1). Exogenous CTS significantly decreased free proline content in leaves under salt stress (Table 1). Pyruvic acid (PA) content increased in response to salt stress, however, no significant difference in PA content between CTS-treated and untreated plants under normal and salt stress conditions was noticed (Table 1). CTS pretreatment did not cause significant change in pyruvate kinase (PK) activity under normal condition, but significantly increased PK activity under salt stress (Table 1).

**Table 1:** Effects of exogenous chitosan (CTS) on changes in sucrose content, fructose content, glucose content, sucrose synthase (SS) activity, sucrose phosphate synthase (SPS) activity, invertase activity, total amino acids (TAA) content, free proline (Pro) content, glutamic acid (Glu) content, γ-aminobutyric acid (GABA) content, pyruvic acid (PA) content, and pyruvate kinase (PK) activity in leaves of creeping bentgrass under normal and salt stress conditions at 24 d. Same letters indicate no significant difference (*n* = 4, and *p* ≤ 0.05). C, control; C + CTS, control pretreated with CTS; S, salt stress; S + CTS, salt-stressed plants pretreated with CTS

Parameters	C	C + CTS	S	S + CTS
Sucrose content (mg g^− 1^ DW)	12.45 + 1.09 d	21.88 + 4.51 c	36.05 + 3.22 b	63.43 + 8.58 a
Fructose content (mg g^− 1^ DW)	9.51 + 1.55 c	3.46 + 0.65 d	28.66 + 0.29 a	23.71 + 0.70 b
Glucose content (μmol g^− 1^ DW)	7.79 + 0.85 c	5.44 + 0.48 c	41.27 + 1.60 a	25.83 + 2.27 b
SS activity (μg min^−1^ mg^− 1^ Prot)	0.89 + 0.06 c	1.01 + 0.06 bc	1.16 + 0.05 b	1.54 + 0.01 a
SPS activity (μg min^−1^ mg^− 1^ Prot)	29.08 + 1.82 c	18.67 + 1.51 c	127.26 + 7.28 a	67.40 + 3.85 b
Invertase activity (μg min^−1^ mg^− 1^ Prot)	627.50 + 12.71 a	643.37 + 12.70 a	425.33 + 10.28 b	353.93 + 9.64 c
TAA content (μmol mg^−1^ Prot)	54.19 + 6.54 c	62.27 + 5.99 c	114.75 + 7.09 b	137.53 + 3.06 a
Free Pro content (μg g^−1^ DW)	1.06 + 0.31 c	0.85 + 0.09 c	4.87 + 0.31 a	3.15 + 0.07 b
Glu content (μg g^−1^ DW)	374.00 + 5.19 b	332.85 + 5.46 c	386.48 + 9.42 b	412.71 + 3.08 a
GABA content (μmol mg^−1^ DW)	821.47 + 18.74 b	895.28 + 10.92 a	414.45 + 9.18 d	536.65 + 20.73 c
PA content (μg mg^−1^)	155.99 + 13.40 b	158.34 + 17.79 b	207.39 + 11.26 a	208.77 + 5.50 a
PK activity (nmol^−1^ min ^− 1^ mg Prot)	299.15 + 7.17 c	280.86 + 6.46 c	635.5 + 19.61 b	689.03 + 12.33 a

### Effects of CTS on physiological changes in root

**Fig. 4:**
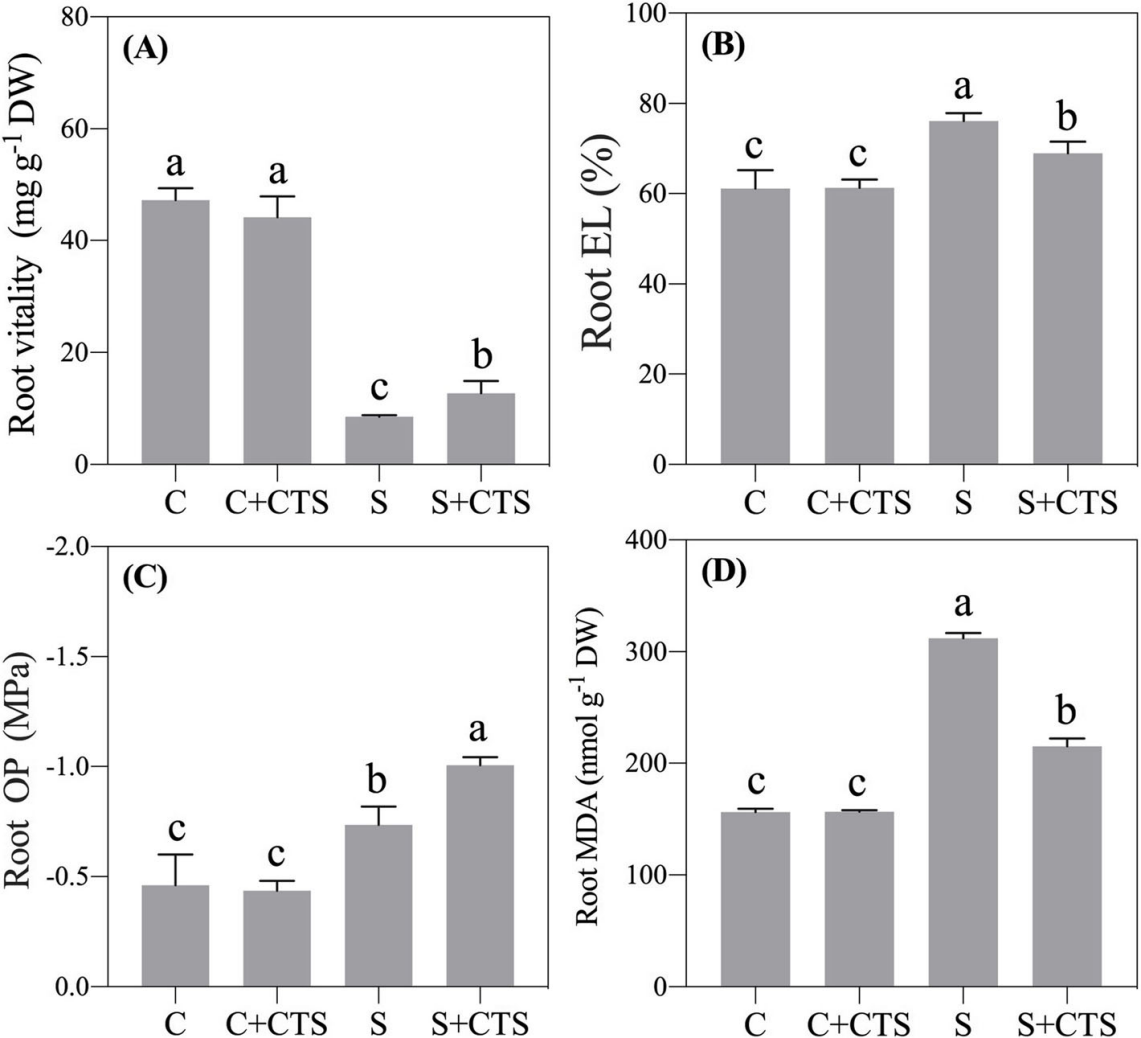
Changes in (**a**) root vitality, (**b**) electrolyte leakage (EL), (**c**) osmotic potential (OP), and (**d**) malondialdehyde (MDA) content in roots of creeping bentgrass affected by the application of chitosan (CTS) under normal and salt stress conditions at 24 d. Bars represent standard errors. Same letters above columns indicate no significant difference (*n* = 4, and *p* ≤ 0.05). C, control; C + CTS, control pretreated with CTS; S, salt stress; S + CTS, salt-stressed plants pretreated with CTS

Under normal condition, exogenous CTS did not significantly affect root vitality, EL, OP, and MDA content in roots (Fig. 4a-d). The root vitality reflects the health status of roots. Successive increasing salt concentration decreased root vitality, but the CTS application significantly alleviated the decline in root vitality(Fig. 4a). Salt stress also significantly decreased OP and increased EL and MDA content in roots (Fig. 4b-d). CTS-pretreated plants showed 13.15% or 31.07% decrease in EL or MDA content in roots than untreated plants, respectively (Fig. 4b and d). For OP in roots, CTS-pretreated plants had lower OP than untreated plants under salt stress (Fig. 4c).

### Effects of CTS on changes of endogenous PAs, Na^+^ content, K^+^ content, and transcript levels of genes involved in Na^+^ transportation in leaf and root

**Fig. 5:**
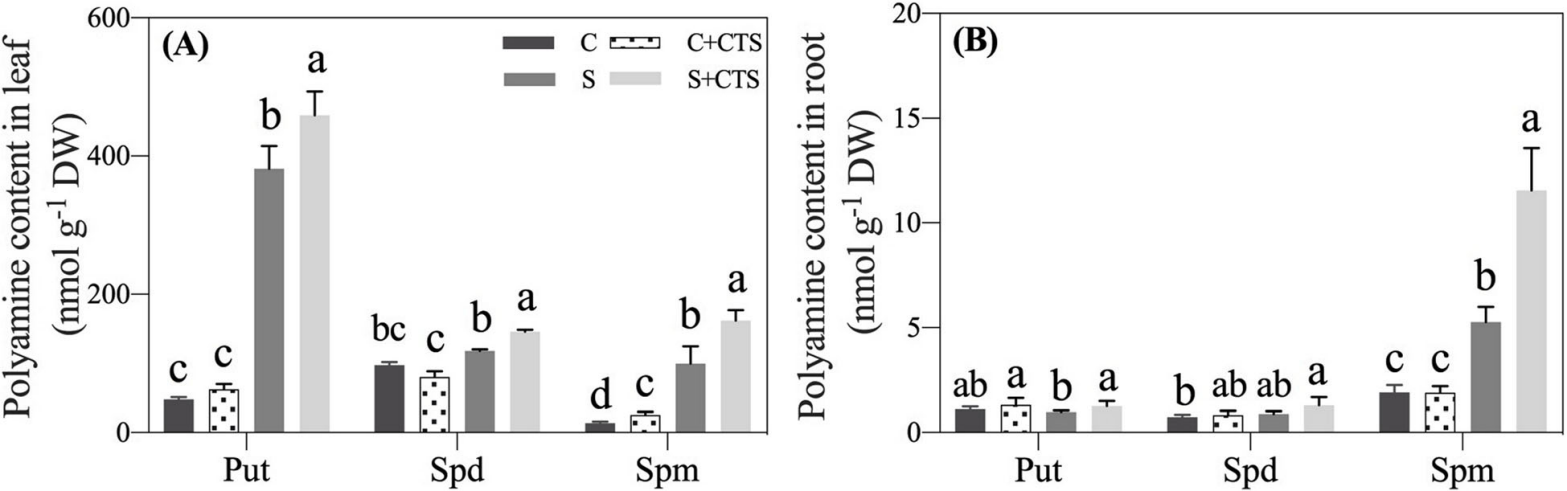
Changes in polyamines content in (**a**) leaves and (**b**) roots of creeping bentgrass affected by theapplication of chitosan (CTS) under normal and salt stress conditions at 24 d. Bars represent standard errors. Same letters above columns indicate no significant difference (*n* = 4, and *p* ≤ 0.05). C, control; C + CTS, control pretreated with CTS; S, salt stress; S + CTS, salt-stressed plants pretreated with CTS

Under normal condition, the level of endogenous putrescine (Put), spermidine (Spd) and spermine (Spm) content in leaves were relatively lower, whereas salt stress significantly increased Put, Spd, and Spm accumulation in leaves (Fig. 5a). CTS-pretreated plants exhibited 20.32%, 23.39%, or 62.78% increase in Put, Spd, and Spm content compared with untreated plants in leaves under salt stress (Fig. 5a). For changes of PAs in roots, CTS-pretreated plants had significantly higher Put content than untreated plants in roots under salt stress (Fig. 5b). There was no significant difference in Spd content between CTS-pretreated and untreated plants in roots under normal condition or salt stress. Spd content in roots only significantly increased in CTS-treated plants in response to salt stress. Salt stress significantly induced Spm accumulation in roots and CTS-pretreated plants had 100% increase in Spm content than untreated plants in roots under salt stress (Fig. 5b).

**Fig. 6:**
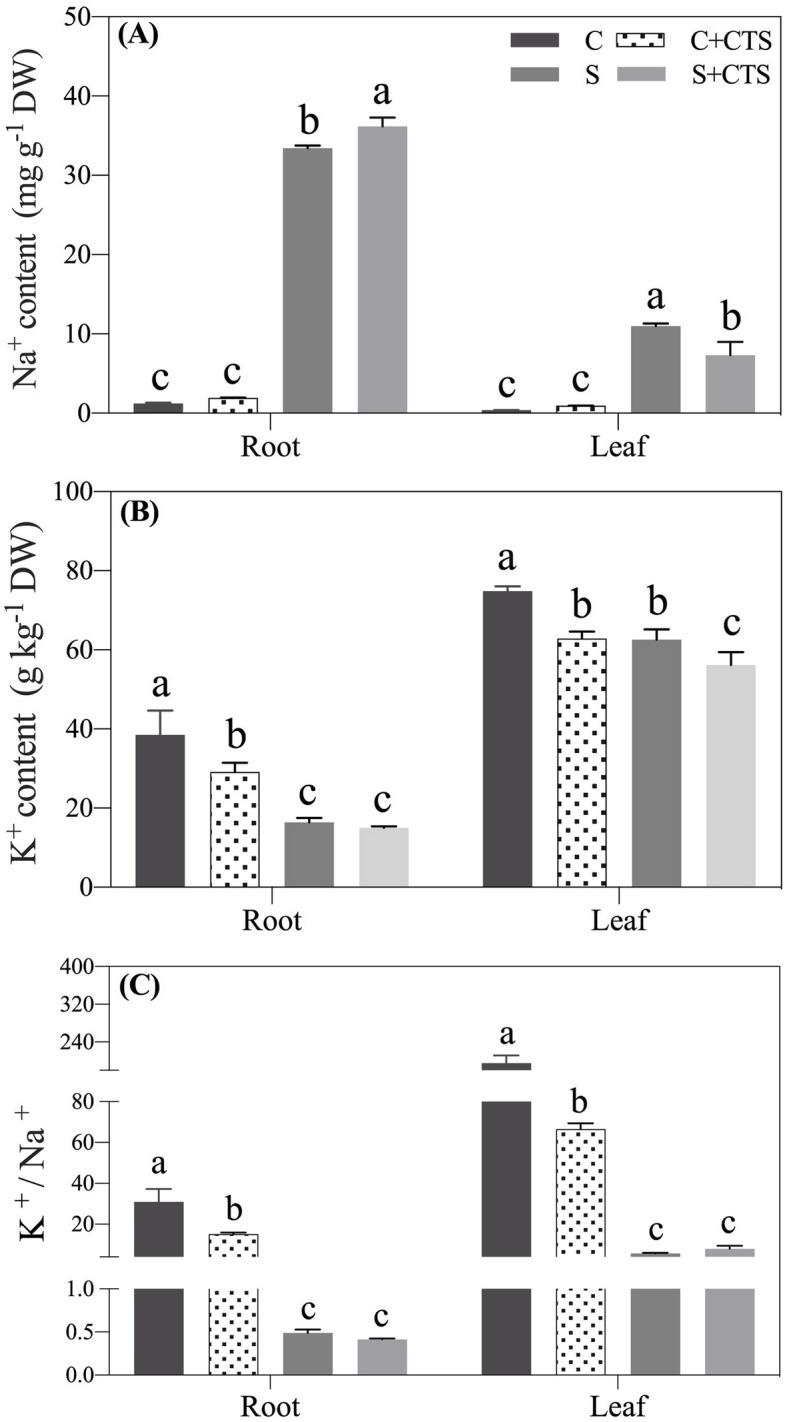
Changes in (**a**) Sodium (Na^+^) content, (**b**) potassium (K^+^) content, and (**c**) K^+^/Na^+^ affected by theapplication of chitosan (CTS) in leaves and roots of creeping bentgrass under normal and salt stress conditions at 24 d. Bars represent standard errors. Same letters above columns indicate no significant difference (*n* = 4, and *p* ≤ 0.05). C, control; C + CTS, control pretreated with CTS; S, salt stress; S + CTS, salt-stressed plants pretreated with CTS

**Fig. 7:**
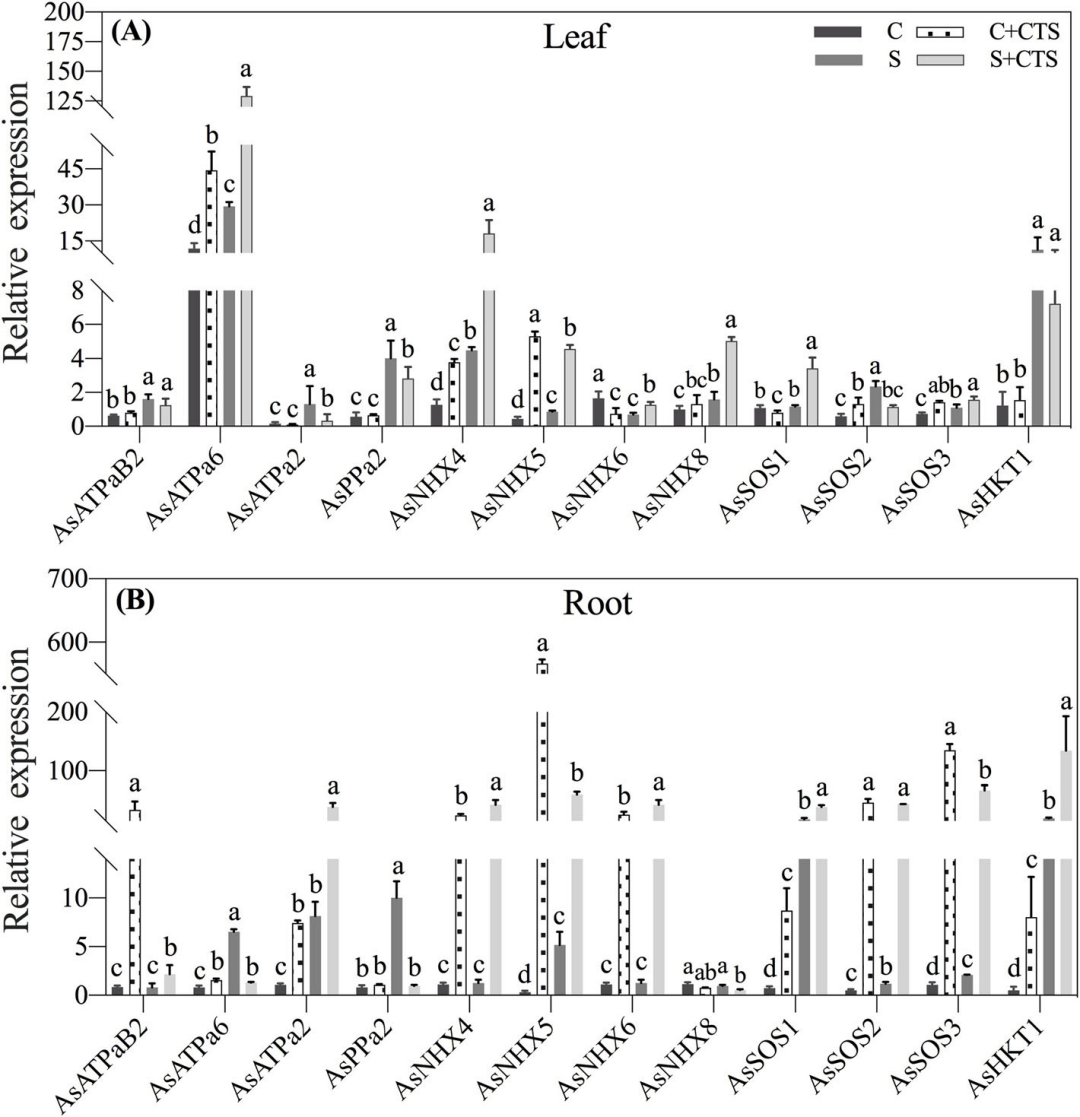
Changes in genes relative expression in (**a**) leaves and (**b**) roots of creeping bentgrass affected by the application of chitosan (CTS) under normal and salt stress conditions at 12 d. Bars represent standard errors. Same letters above columns indicate no significant difference (*n* = 4, and *p* ≤ 0.05). C, control; C + CTS, control pretreated with CTS; S, salt stress; S + CTS, salt-stressed plants pretreated with CTS

**Fig. 8:**
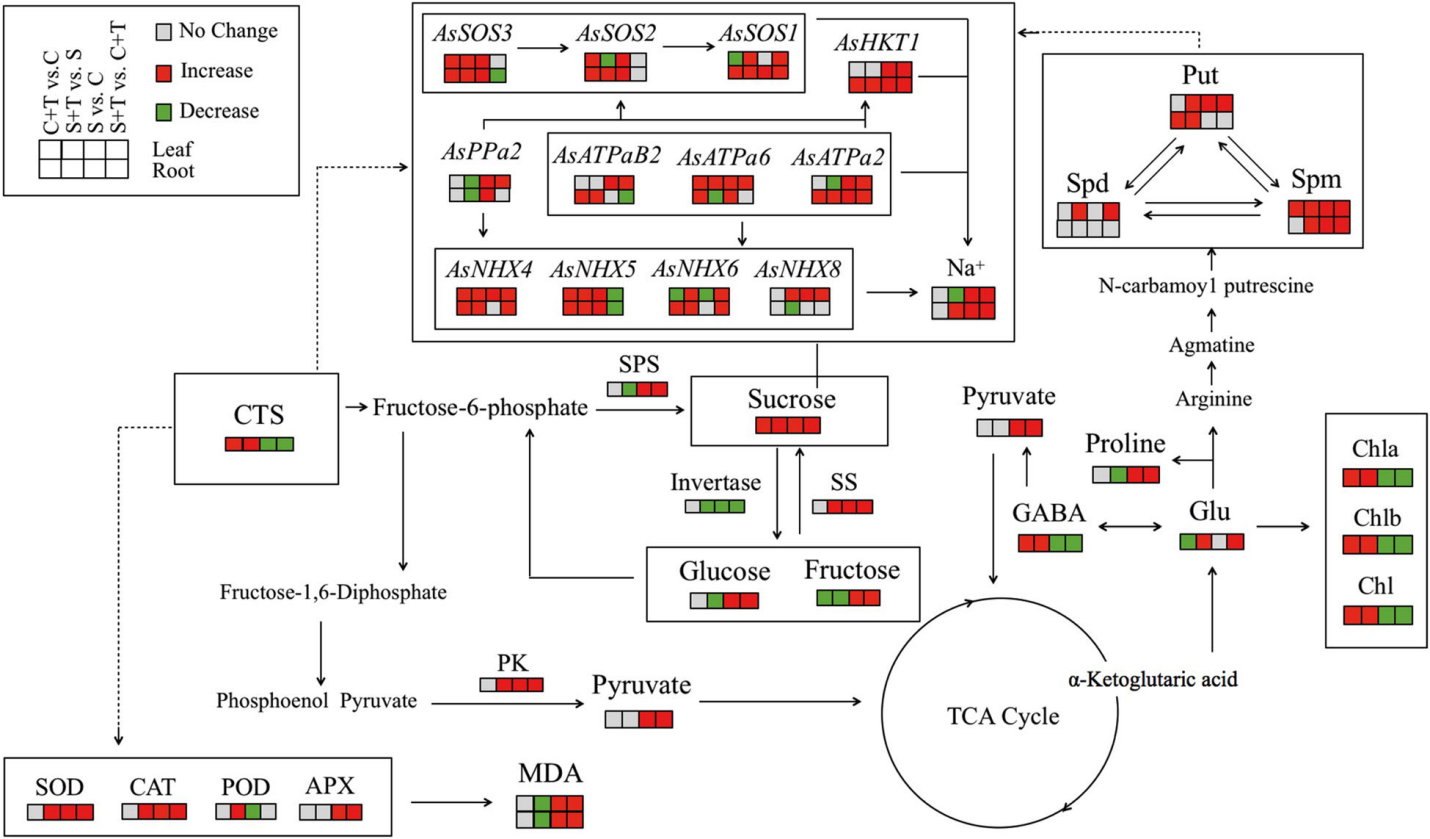
Proposed key pathways involved in immanent and CTS-regulated adaptive response to salt stress in perennial creeping bentgrass

Under normal condition, the Na^+^ content was maintained at a very low level in leaves and roots, and CTS pretreatment had no effect on the Na^+^ content (Fig. 6a). Salt stress significantly increased Na^+^ content in roots and leaves. CTS-pretreated plants accumulated significantly higher Na^+^ in roots, but lower Na^+^ in leaves than untreated plants under salt stress (Fig. 6a). The K^+^ content and K^+^/Na^+^ ratio were significantly higher in CTS-untreated plants than those in CTS-pretreated plants in leaves and roots under normal condition (Fig. 6b and c). Exogenous CTS pretreatment had significant effects on K^+^ content in leaves under salt stress but no significant effects on K^+^ content in roots and K^+^/Na^+^ ratio in roots and leaves under salt stress (Fig. 6b and c). For changes of genes expression involved in Na^+^ transport in leaves, exogenous CTS had no significant effects on genes encoding *AsATPaB2*, *AsATPa2*, *AsPPa2*, *AsNHX8*, and *AsHKT1*, but significantly up-regulated *AsATPa6*, *AsNHX4*, *AsNHX5*, and *AsSOS2* under normal condition (Fig. 7a). Under salt stress, CTS-pretreated plants showed significantly higher expression levels of *AsATPa6*, *AsNHX4*, *AsNHX5*, *AsNHX6*, *AsNHX8*, *AsSOS1*, and *AsSOS3* than untreated plants in leaves (Fig. 7a). Salt stress significantly up-regulated *AsATPa6*, *AsATPa2*, *AsPPa2*, *AsNHX5*, *AsSOS1*, *AsSOS3*, and *AsHKT1* in roots (Fig. 7b). Under normal condition, exogenous CTS application induced significant increases in *AsATPaB2*, *AsATPa6*, *AsATPa2*, *AsNHX4*, *AsNHX5*, *AsNHX6*, *AsSOS1*, *AsSOS2*, *AsSOS3*, and *AsHKT1* in roots. Under salt stress, *AsATPaB2*, *AsATPa6*, *AsATPa2*, *AsNHX4*, *AsNHX5*, *AsNHX6*, *AsSOS1*, *AsSOS2*, *AsSOS3*, and *AsHKT1* were significantly up-regulated by exogenous CTS in roots (Fig. 7b). Figure 8 showed proposed key pathways involved in immanent and CTS-regulated adaptive response to salt stress in perennial creeping bentgrass.

## Discussion

High soil salinity severely reduces water uptake leading to physiological drought, thereby inhibiting plant growth [35]. It has been well documented that maintenance of higher osmotic adjustment (OA) capacity and WUE contributed to better water status in plants under salt stress [36]. In the current study, salt stress significantly decreased OP in leaves (Fig. 1d) and roots (Fig. 4c) that could be a positive response for acclimating to salt damage in creeping bentgrass. CTS-pretreated creeping bentgrass maintained significantly higher WUE and lower OP in leaves (Fig. 1d-e), which could be associated with better water status in plants under salt stress. Our recent study also found that exogenous GABA application effectively alleviated salt-induced damage through enhancing OA and WUE in creeping bentgrass [37]. When plants are exposed to salt stress, accelerated oxidative damage and water deficit will cause photoinhibition [38]. Successive increasing salt concentration significantly decreased Chl content, Pn, Fv/Fm, and PIABS in leaves of creeping bentgrass (Fig. 2). However, CTS-pretreated creeping bentgrass had significantly higher Chl content, Pn, PIABS, and Fv/Fm than untreated plants, which reflected better photosynthetic capacity in CTS-pretreated plants under salt stress in this study. In addition, the application of CTS also improved plant growth and Chl content under normal condition. It has been reported that the CTS could be used as an exogenous plant growth regulator for improving growth and quality of crops [27]. Thus, dual positive functions of CTS in regulating growth under normal condition and stress tolerance highlight its importance in agricultural cultivation and production.

Salt stress increases accumulation of reactive oxygen species (ROS) such as superoxide radical and hydrogen peroxide (H_2_O_2_) that are responsible for oxidative damage and ultimately cell death [5]. Improvement or maintenance of higher antioxidant enzyme activities such as SOD, CAT, POD, and APX is important for reducing oxidative damage in different plant species under salt stress [39–41]. Current results found that exogenous CTS played an important role in reducing lipid peroxidation in association with significant increase in antioxidant enzyme (SOD, CAT, and POD) activities in creeping bentgrass under salt stress (Fig. 3), which was consistent with previous study about the effect of CTS on improving the salt tolerance in sunflower seedlings through enhancing CAT and POD activities. As the primary organ for water and nutrition intake, root system is easily damaged by high soil Na^+^ [42]. Successive increasing salt concentration seriously reduced root vitality (Fig. 4a), whereas CTS application effectively alleviated the negative effect in creeping bentgrass. These phenotypic and physiological changes in roots and leaves indicated positive effects of CTS on alleviating salt stress damage in creeping bentgrass.

Regulation of sugar metabolism plays an essential role for plants acclimation to environmental stress [43]. Exogenous application of CTS significantly increased endogenous CTS content in leaves of creeping bentgrass (Fig. 1b). Based on metabolic pathways, the CTS could be converted directly to other sugars and pyruvate that participates in the tricarboxylic acid cycle and the biosynthesis of glutamic acid and other amino acids (Fig. 8). When plants are subjected to abiotic stress, soluble sugars such as fructose, glucose, and sucrose accumulate in cells as important signaling molecules for stress signal transduction, osmolytes for OA, and energy substances for energy supply [44, 45]. Sucrose is a major photoassimilate and can be transferred from source leaves to roots. Invertase catalyzes sucrose degradation into glucose and fructose for cell biosynthesis and metabolism [46]. Sucrose synthesis can be catalyzed by SPS from fructose-6-phosphate or transformed from glucose and fructose by SS. In this study, salt stress significantly improved sucrose synthesis through activating SPS and SS activities and reducing invertase activity (Table 1). Interestingly, exogenous CTS application further increased salt-induced sucrose accumulation that could be associated with higher SS activity and lower invertase in leaves of creeping bentgrass. In an earlier study, it has been found that sucrose accumulation was conducive to PSII protection in plants under salt stress [47]. The study of Schwender et al. [48] demonstrated that 90% glucose could be converted to pyruvate by glycolysis in *Brassica napus*. PK is one of main rate-limiting enzymes in the glycolysis process for energy production [49, 50]. CTS-improved PK activity might enhance conversion of CTS, glucose, and fructose into pyruvate for energy metabolism in creeping bentgrass (Table 1), but the mechanism underlying changes of sugars and metabolic enzymes still need to be further investigated during salt stress.

Amino acids accumulation and metabolism are important for plants to deal with stress damage [51]. Proline has been extensively studied as a stress-responsive amino acid and its function is involved in OA and redox balance in plants during abiotic stress [52, 53]. Glu is a main metabolite of nitrogen metabolism in plants associated with the maintenance of carbon-nitrogen balance, other amino acid metabolism, Chl biosynthesis during normal growth and stress response [54]. Increasing evidences indicate that GABA is involved in regulating tolerance to abiotic stress through enhancing OA, antioxidant defense, and metabolic balance in plants [55]. Previous study has shown that amino acid synthesis pathway was significantly enhanced in barley (*Hordeum vulgare*) leaf under salt stress, and amino acids such as Glu and GABA were key metabolites involved in salt stress response [56]. Exogenous GABA induced salt tolerance associated with increase in sugars (glucose, fructose, maltose, and trehalose) and amino acids (Glu, alanine, leucine, valine, asparagine, lysine, threonine, and cysteine) content in leaves of creeping bentgrass [37]. In the current study, CTS-pretreated creeping bentgrass exhibited significantly higher TAA, Glu, and GABA content than untreated plants under salt stress (Table 1), which could explain why CTS-pretreated plants had better OA capacity, water status, and metabolic homeostasis as compared to untreated plants. However, the application of exogenous CTS alleviated salt stress-induced increase in proline accumulation in leaves (Table 1). The proline accumulation is also often regarded as an indicator of stress damage. More severe stress damage, more proline accumulation in plants under salt or other abiotic stress [37, 57, 58].

Many studies have reported that increased PAs level is an important adaptive response when plants are subjected to salt stress [59–61]. PAs-regulated salt tolerance could be associated with activation of SOS signaling pathways, nitrogen metabolism, and antioxidant defense system to maintain ions and metabolic balance in plants under salt stress [62]. A previous study found that the salt-tolerant barley cultivar 'J4′ accumulated significantly higher endogenous Spd and Spm than salt-sensitive 'KP7′ in response to successive increasing salt concentration [60]. The CTS can be converted into pyruvate involved in the tricarboxylic acid cycle for the production of glutamic acid which can be metabolized to arginine for the synthesis of PAs (Fig. 8). It has been found that exogenous CTS significantly improved endogenous Put, Spd, and Spm accumulation contributing to enhanced drought tolerance in white clover (*Trifolium repens*) [28, 63]. In this study, Put, Spd, and Spm accumulated significantly in leaves and Spm increased considerably in roots of creeping bentgrass under salt stress (Fig. 5). Exogenous CTS application further promoted salt-induced PAs accumulation in leaves and roots of creeping bentgrass. These findings suggested that CTS-regulated salt tolerance could be involved in PAs synthesis in creeping bentgrass.

High Na^+^ levels are generally considered to be the primary factor in salt toxicity in plants [64]. In a saline environment, a possible survival strategy for plants is to effectively isolate excess Na^+^ in roots and to inhibit the transfer of Na^+^ from roots to shoots [65]. Our results showed that the creeping bentgrass with CTS application had significantly higher Na^+^ content in roots, but lower Na^+^ accumulation in leaves as compared to the plants without CTS pretreatment (Fig. 6). Analyses of genes expression also found that salt stress significantly up-regulated the *AsHKT1* expression in leaves and roots. More importantly, exogenous CTS could further enhance the *AsHKT1* expression in roots of creeping bentgrass, which indicated that CTS could regulate *AsHKT1* to isolate Na^+^ in roots and to inhibit Na^+^ transport to leaves during a long period of salt stress. The CTS pretreatment also significantly activated the SOS pathway in roots under salt stress (Fig. 7). The study of Wang et al. (2014) found out that main function of *AtSOS1* is to extrude Na^+^ from the cytosol into the rhizosphere in *Arabidopsis thaliana* under the normal K^+^ plus salt stress. However, the *AtSOS1* regulated the long-distance transport of Na^+^ from roots to leaves in *Arabidopsis thaliana* under the low K^+^ plus salt stress [66]. In current study, the CTS-activated SOS pathway could be involved in Na^+^ excretion, since the creeping bentgrass was subjected to salt stress with enough K^+^ supply in Hoagland’ solution. In addition, the separation of Na^+^ into vacuoles is considered as a key mechanism to avoid toxic effects of Na^+^ in the cytoplasm. The Na^+^ compartmentalization also can provide additional osmotic adjustment for water maintenance under salt stress [17, 67]. The CTS-treated creeping bentgrass (S + CTS) had significantly higher expression levels of *AsNHX4*, *AsNHX5*, and *AsNHX6* than untreated plants (S) in roots and leaves under salt stress (Fig. 7), suggesting that the CTS could enhance the capacity of Na^+^ compartmentalization in cells. Interestingly, the CTS significantly up-regulated *AsATPaB2* and *AsATPa2* in roots and *AsATPa6* in leaves, which could be related to enhanced proton motive force in creeping bentgrass under salt stress. A previous study has proved that the inhibition of Spd and Spm biosynthesis decreased tonoplast H^+^-ATPase activities resulting in significant decrease in salt tolerance of barley seedlings [60]. It is worth further study whether PAs are involved in CTS-regulated Na^+^ transport in plants during salt stress.

## Conclusion

The addition of exogenous CTS is a cheap and effective measure to alleviate growth inhibition and stress damage caused by salt stress in creeping bentgrass. In response to salinity, exogenous CTS increased antioxidant enzyme activities, thereby reducing oxidative damage to roots and leaves. CTS-induced increase in sucrose and GABA accumulation and metabolism played important role in OA and energy metabolism during stress. The CTS also regulated Na^+^ transport though increasing *AsHKT1* expression for inhibiting Na^+^ transport to photosynthetic tissues, enhancing SOS pathway associated with Na^+^ excretion from cytosol into rhizosphere, and up-regulating the expression of *AsNHX4*, *AsNHX5*, and *AsNHX6* involved in Na^+^ compartmentalization from cytoplasm into vacuoles in roots and leaves under salt stress. In addition, CTS-induced PAs accumulation could be an important regulatory mechanism contributing to enhanced salt tolerance in creeping bentgrass. These findings reveal important functions of CTS on regulating Na^+^ transport, enhancing sugars and amino acids metabolism, and increasing PAs accumulation, which contributes to the alleviation of salt stress in perennial creeping bentgrass. To further understand the CTS-induced salt tolerance in plants, future studies will focus on investigating changes in global metabolites based on metabolomics and analyzing possible signal transduction pathways involved in GABA-regulated PAs accumulation, antioxidant defense, and Na^+^ transport under salt stress.

## Methods

### Plant materials and treatments

Seeds of creeping bentgrass (cv. PA-1) were purchased from Tee-2-Green Company (Oregon, USA) and used as a plant material. Seeds (3.8 g/m^2^) were sown in seedling pots filled with sterilized quartz sand and germinated in a plant growth chamber (photoperiod cycle of 10/14 h light/dark, 21/18 °C day/night, 65% relative humidity, and 700 μmol m^− 2^·s^− 1^ PAR) for 8 days. Seedlings were then grown in Hoagland’ solution for 20 days [68]. 28-day-old plants were pretreated with or without 0.5 g/L CTS for 4 days. The CTS or NaCl was dissolved in Hoagland’ solution. The effective dose of CTS was selected based on a preliminary test with a range of concentrations (0.1, 0.2, 0.5, 1, and 2 g/L) for the most effective dose on phenotypic changes. The CTS-pretreated and untreated plants were subjected to NaCl-induced salt stress for 24 days. For salt stress, plants were grown in 100 mmol/L NaCl solution for 4 days, 150 mmol/L NaCl solution for another 4 days, and 200 mmol/L NaCl for 16 days. All solutions were refreshed every day. Four biological replicates were set for each treatment. Plants were sampled at 0, 12, and 24 d of salt stress, respectively, and 4 biological replicates were used to estimate all parameters.

### Physiological measurements

For the estimation of EL, the method of Blum and Ebercon was used [69]. RWC was determined according to the method of Barrs and Weatherley [70]. For OP of leaf and root, fresh leaves or roots were immediately immerged in distilled water for 12 h at 4 °C. After being blotted dry, leaves or roots were frozen in liquid nitrogen for 10 min, and then thawed for 30 min at 4 °C. Leaves or roots were pressed to get cell fluid. The osmolality of cell fluid was measured by using a vapor pressure osmometer (Wescor, Logan, UT, USA), and the OP was converted based on -c × 2.58 × 10^− 3^ [71]. For root viability, the method of McMichael and Burke was used [72]. The determination of Chl content was performed according to the method of Arnon et al. [73]. The Fv/Fm and PIABS were recorded by a Chl fluorescence system (Pocket PEA, Hansatech, the United Kingdom). Leaves were placed in dark for 30 min with leaf clips before analysis. Pn and WUE were measured using a portable photosynthetic system (CIRAS-3, PP Systems, USA) that provided 400 μl L^− 1^ CO_2_ and 800 μmol photon m^− 2^ red and blue light.

### Measurements of total antioxidant capacity and antioxidant enzyme activities

Fresh leaves (0.2 g) were ground on ice with 1.5 mL of 50 mM cold phosphate buffer (pH 7.8) and centrifuged at 12000 g for 30 min at 4 °C. The supernatant was used for the determination of antioxidant enzyme activities and MDA content. Activity of SOD, CAT, POD, or APX was measured by recording changes in absorbance at 560, 240, 470, or 290 mm, respectively [74–76]. Protein content was measured according to the method of Bradford [77]. For MDA content, 0.5 mL of the supernatant was mixed with 1 mL of reaction solution containing 20% trichloroacetic acid and 0.5% thiobarbituric acid (TBA). The mixture was heated in a water bath at 95 °C for 15 min and then rapidly cooled in an ice bath. The solution was centrifuged at 8000 g for 10 min at 4 °C. The absorbance of supernatant was recorded at 532, 600 and 450 nm [28].

### Measurements of sugars, amino acids, and enzyme activities related to sugars and energy metabolism

The CTS content (Art. No. ml020132) was determined by using a Test Kit (mlbio Good elisakit producers, Shanghai, China) according to the manufacturer’s instructions. Pyruvate kinase (PK) activity (Art. No. PK-2-Y), pyruvic acid content (Art. No. PA-1-Y), sucrose synthase (SS) activity (Art. No. SSII-1-Y), sucrose phosphate synthase (SPS) activity (Art. No. SPS-1-Y), hexokinase activity (Art. No. HK-2-Y), invertase activity (Art. No. ZTM-1-Y), sucrose content (Art. No. ZHT-2-Y), glucose content (Art. No. PT-2-Y), and fructose content (Art. No. GT-2-Y)were determined by using Test Kits (Suzhou Comin Biotechnology, Suzhou, China) according to manufacturer’s instructions. For free proline content, fresh leaves (0.2 g) were ground with 3% aqueous sulfosalicylic acid and then centrifuged at 10000 g for 10 min to obtain supernatant. The reaction mixture (1 mL of the supernatant, 1.5 mL of acid ninhydrin, and 1.5 mL of glacial acetic acid) was boiled for 30 min. After being cooled at room temperature, the reaction mixture was extracted with five mL of benzene and the absorbance was recorded at 520 nm [78].

### Measurement of endogenous polyamine and Na^+^ / K^+^ content

For the measurement of endogenous PAs content, the methods of Duan [79] and Li [80] were used. The Na^+^ or K^+^ content was detected by using a flame atomic absorption spectrophotometer (Analytik Jena AG, Jena, Germany) and the assay method has been recorded in our previous study [37].

### Genes expression analyses

For determining effects of CTS on changes in selected gene transcript levels, real-time quantitative polymerase chain reaction (qRT-PCR) was used. The Rneasy Mini Kit (Qiagen, Duesseldorf, Germany) was used for extracting total RNA in fresh leaves or roots. The RNA was reverse-transcribed to cDNA using a revert Aid First Stand cDNA Synthesis Kit (Fermentas, Lithuania). Primers of sodium-hydrogen antiporter genes (*AsNHX4, AsNHX5, AsNHX6,* and *AsNHX8*), H^+^ transporter genes (*AsATPaB6*, *AsATPa6*, *AsATPa2*, and *AsPPa2*), salt overly sensitive genes (*AsSOS1*, *AsSOS2*, and *AsSOS3*), high-affinity Na^+^/K^+^-permeable transporter (*AsHKT1*), and reference gene (*β-actin*) were given in Table S1. For all genes, PCR conditions (iCycler iQ qRT-PCR detection system with SYBR Green Supermix, Bio-Rad, USA) were as follows: 5 min at 94 °C and 30 s at 95 °C (45 repeats of denaturation), annealing and extending 45 s at 62 °C, and amplicon from 60 to 95 °C to obtain the melting curve. Transcript levels of all genes were calculated according to the formula 2^−∆∆Ct^ described by Livak and Schmittgen [81].

### Statistic

The SPSS 23 (IBM, Armonk, NY, USA) was used for analyzing all data. Significant differences among C, C + NaCl, CTS, and CTS + NaCl treatments were tested by using the least significant difference (LSD) at *p* ≤ 0.05.

## Supplementary Information

**Additional file 1 Table S1.** Primer sequences of gene.

## Data Availability

The data used and/or analyzed during the current study are available from the corresponding author on reasonable request.

## Abbreviations

CTS: Chitosan
Na^+^: Sodium ions
ROS: Reactive oxygen species
H_2_O_2_: Hydrogen peroxide
SOS: Salt overly sensitive pathways
NHX: Na^+^/H^+^ antiporters
K^+^: Potassium ions
PAs: Polyamines
RWC: Relative water content
FW: Fresh weight
SW: Saturated weight
DW: Dry weight
WUE: Water use efficiency
OP: Osmotic potential
Chl: Total chlorophyll
Fv/Fm: Photochemical efficiency
PIABS: Performance index on absorption basis
Pn: Net photosynthetic rate
MDA: Malondialdehyde
EL: electrolyte leakage
SOD: Superoxide dismutase
CAT: Catalase
POD: Peroxidase
APX: Ascorbate peroxidase
SS: Sucrose synthase
SPS: Sucrose phosphate synthase
TAA: Total amino acids
Pro: Free proline
Glu: Glutamic acid
ROS: Reactive oxygen species
GABA: γ-Aminobutyric acid
PA: Pyruvic acid
PK: Pyruvate kinase
Put: Putrescine
Spd: Spermidine
Spm: Spermine
OA: Osmotic adjustment
