# Supplementary material for: Chitosan regulates metabolic balance, polyamine accumulation, and Na+ transport contributing to salt tolerance in creeping bentgrass
Source: BMC Plant Biol. 2020 Nov 4;20:506. doi: 10.1186/s12870-020-02720-w (PMC7640404; doi:10.1186/s12870-020-02720-w)
Supplement: Supplementary file 1 — Additional file 1 Table S1. Primer sequences of gene. [file 12870_2020_2720_MOESM1_ESM.docx]

**Table S1.** Primer sequences of genes.

| Targetgene | Forward Primer (5′-3′) | Reverse Primer(5′-3′) |
| --- | --- | --- |
| *AsATPa B2* | CTCAGCAGCACGTGAAGAGG | ATACGCCCAGCTCGCTCATA |
| *AsATPa6* | GGATTCTCGCCGCTTGGATTGG | GGTGTAGTGGAGCAGGTTGTTGTC |
| *AsATPa2* | CCGTCATCATTGCCGTCGTCTAC | GTCAGCCTTGGAGCGGTTCTTG |
| *AsPPa2* | GGTGTGGCGGTCGTTGATGTC | GGCATAGTCAGGCTTGGCAGTTC |
| *AsNHX4* | CCACTCTGGTGTTTGGTTTCCTGAC | TCCGCTCCCTGACGCTTCTTC |
| *AsNHX5* | GCACCGCATCATTTACAGCACTG | GCTCATCTTGTTCGTCATCCTCTCC |
| *AsNHX6* | CGCTGGAGCTGAGCATGAACC | TGGCCGAGCACGAAGGAGAG |
| *AsNHX8* | CAACGCACGAGACGAGGCTTC | GGTTGATGGCAGCAGCACTCC |
| *AsSOS1* | ACTGCCTGCTGATGTTCGTGATC | TGAATGCCTGGTGCTTAGTCTCTG |
| *AsSOS2* | GGATTAGGCTCTGCTCAGGTGTTG | TGCCGCTGGTCCTGGTAGTG |
| *AsSOS3*  *AsHKT1*  *β-Actin* | GGCGTCGTGCTCAACCAGTTC  TACTACCACCTGCTCTCGGC  CCTTTTCCAGCCATCTTTCA | CGAGCGTTGACAAGAGAACCTGAG  AACTTCTCCCACGGGCTCAT  GAGGTCCTTCCTGATATCCA |
